# Low-Velocity Impact of Clamped Rectangular Sandwich Tubes with Fiber Metal Laminated Tubes

**DOI:** 10.3390/polym16131833

**Published:** 2024-06-27

**Authors:** Yao Wang, Jianxun Zhang, Hui Guo, Hui Yuan

**Affiliations:** 1 Shock and Vibration of Engineering Materials and Structures Key Lab of Sichuan Province, Southwest University of Science and Technology, Mianyang 621010, China; 18563226065@163.com (Y.W.); guohui56789@126.com (H.G.); 2 State Key Laboratory for Strength and Vibration of Mechanical Structures, School of Aerospace Engineering, Xi’an Jiaotong University, Xi’an 710049, China; 13032499002@163.com; 3 Key Laboratory of Mechanics on Disaster and Environment in Western China Attached to the Ministry of Education of China, Lanzhou University, Lanzhou 730000, China

**Keywords:** dynamic response, rectangular sandwich tube, FML tube, low-velocity impact

## Abstract

Fiber metal laminated sandwich tubes are made up of alternating fiber-reinforced composite and metal layers. Fiber metal laminated tubes have the advantages of the high strength and high stiffness of fiber and the toughness of metal, so they have become an excellent load-bearing and energy-absorbing, lightweight structure. Due to the complexity of the fiber layup, it is difficult to establish an analytical model of the relevant structural properties. In this work, introducing the number and volume fraction of fiber layup, based on the modified rigid–plastic model, an analytical model is established for low-velocity impacts on sandwich tubes with fiber metal laminated tubes, which provided a theoretical basis for the design of fiber–metal composite tubes. In addition, a numerical simulation was conducted for low-velocity impacts on clamped rectangular sandwich tubes with fiber metal laminated (FML) tubes and a foam core. By comparing the results obtained from the theoretical analysis and numerical calculations, it is shown that the analytical results can reasonably agree with the numerical results. The influences of the metal volume fraction (MVF), the strength ratio factor of the FML metal layer to the FML composite layer, and the relative strength of the foam on the dynamic response of the rectangular sandwich tubes with FML tubes and a metal foam core (MFC) are discussed. It is shown that by increasing the fiber content and fiber strength of the FML tubes and the foam strength, the load-carrying and energy-absorbing capacity of the rectangular sandwich tubes can be effectively improved, especially by changing the fiber properties. In addition, present analytical solutions can be applied to make predictions about the dynamic response of the rectangular sandwich tubes with FML tubes and MFC during impacts with low-velocity and reasonably heavy-mass.

## 1. Introduction

Fiber metal laminated (FML) tubes are made up of alternating fiber-reinforced composite and metal layers, and the composite layer is made of polymer materials (such as glass fiber- or carbon fiber-reinforced fibers) and metal alloy materials [1]. FML has excellent properties that combines the properties of metals and composite materials, such as good impact resistance and plasticity, good mechanical adaptability, and excellent fatigue properties, as well as low weight [2]. A sandwich tube with a metal foam core (MFC) is a typical lightweight tube consisting of rigid and strong inner and outer tubes and MFC sandwiched between the inner and outer tubes. Due to its good energy absorption and impact resistance, as well as high specific strength and specific stiffness, sandwich tubes with MFC have been used in many fields such as aviation, space, navigation, high-speed rail, and so on [3,4,5,6,7,8,9,10,11,12,13,14,15,16,17,18,19,20]. Sandwich tubes designed with FML tubes and MFC provide more choice for energy-absorbing structures. Therefore, there is a need to investigate low-velocity impacts on sandwich tubes with FML tubes and MFC.

In the past decade or so, the mechanical behavior of sandwich tubes under quasi-static loading has been extensively studied. Zhang et al. [21] established the yield criterion and developed an analytical model to predict the plastic behavior of fully clamped slender rectangular sandwich tubes with MFC. Liu et al. [22] studied the mechanics of sandwich tubes under lateral loading by a quasi-static transverse indentation test and finite element calculations, and found that the relative differences in denting characteristics between the inner and outer tubes are related to the material and geometric properties of the tubes and the interactions between the layers. Guelou et al. [23] experimentally investigated the static compression of wood-based sandwich tubes, and found the energy absorption of such structures under static conditions as well as coupling effects. Niknejad et al. [24] investigated the indentation behavior of laminated and composite tubes with agglomerated cork cores under transverse loading by a cylindrical indenter experimentally, and found that cork-filled composite tubes absorb more total energy. Baroutaji et al. [25] conducted a systematic investigation on the lateral fragmentation of sandwich tubes with circular tubes and MFC under quasi-static loading by means of experimental and numerical methods, and found that a tube with a small inner diameter and larger foam thickness would be better suited for use as an energy-absorbing component. Kim et al. [26] investigated the extrusion behavior of square composite sandwich tubes with woven fabric carbon/epoxy skins and aluminum honeycomb cores by means of quasi-static and dynamic shock tests, and concluded that the dynamic crushing modes collapsed in a more stable manner than the corresponding static tests. Fan et al. [27] conducted a systematic study of thin-walled circular tubes sandwiched with MFC under quasi-static transverse extrusion through experiments and numerical simulations, and found that the compressive strength and energy absorption of the sandwiched tubes are greater than the sum of the components, and sandwich tubes are more weight-efficient than empty tubes. Shen et al. [28] used experimental, theoretical, and numerical methods to study the transverse crushing response of sandwich tubes with shot aluminum foam, and concluded that the bonding between the tubes and the core of the sandwich tubes varies in different crushing modes. Wu et al. [29] studied the crushing behavior of a sandwich tube with a meta-honeycomb core with zero Poisson’s ratio under transverse and axial compressive loadings experimentally, and concluded that it has a stable crushing curve and crushing mode, and is more stable and efficient than thin-walled composite tubes in energy absorption. Wang et al. [30] investigated the axial and transverse crushing characteristics of sandwich tubes filled with plate-lattice in square tubes by means of experimental and numerical methods, and the findings indicated that the filling of plate-lattice can significantly improve the load carrying capacity and specific energy absorption of the sandwich tubes.

In addition, the dynamic bending behavior of sandwich tubes has been investigated. Zhang et al. [31] obtained an analytical solution for dynamic response of fully clamped rectangular sandwich tubes with MFC under low-velocity impact. Zhang and Guo [32] obtained an analytical solution for the dynamical response of the clamped rectangular sandwich tube with MFC under lateral loads, which was shown to be in good agreement with the numerical results. Guo et al. [33] experimentally investigated the splitting and curling behaviors of square sandwich tubes with MFC subjected to axial low-velocity impact. The deformation modes and load-displacement curves were obtained, and the analytical solution can be used to predict the steady-state load of square sandwich tubes subjected to axial low-velocity impact. Zhang et al. [34] used experimental, theoretical and numerical methods to investigate the dynamical response and energy-absorption characteristics of metal foam-filled circular sandwich tubes under interior blast loads, and observed that circular sandwiched tubes with gradient cores of negative gradient have the optimal resistance. Guelou et al. [35] investigated the dynamic crushing of sandwich tubes with tubes made of carbon or glass fibers and a poplar ply core through experiments, and the results showed that the wood makes a significant contribution tostructural response. Shen et al. [36] conducted a systematic investigation on the dynamic behavior of sandwich tubes under internal explosive loading by both experimental and numerical analyses, and obtained an analytical solution for the dynamic response of the sandwich tube under blast loads. Wang et al. [37] studied the dynamic response of density-graded sandwich tubes with MFC under internal explosive loading through finite element calculation, and concluded that the blast resistance decreases as the density gradient of the core increases.

With the demand for FML tubes with good load-carrying capacity, impact resistance, and energy absorption, more and more attention has been focused on the study of the quasi-static behavior and impact response of FML tubes. Mansor et al. [38] studied the crashworthiness of thin-walled FML tubes in axial compression by means of experiment and numerical calculation, and discovered that FML tubes can be considered as a suitable candidate structure for lightweight energy-absorbing applications with considerable impact resistance. Ahmad et al. [39] conducted a systematic investigation on the impact characteristics and energy absorption of a thin-walled FML tube under axial impact loads by means of an experimental and numerical calculation, and concluded that it can withstand larger impact loading, absorb higher energy, and is more suitable as an impact energy absorber. Subbaramaiah et al. [40] conducted a study of the axial crushing behavior of glass laminate aluminum reinforced epoxy (GLARE) top-hat structures experimentally and numerically, and the results showed that the crushing response of the GLARE top-hat structures is preferable to that of the bare metal equivalent. Song et al. [41] analytically investigated the dynamic response of rectangular sandwich tubes with FML tubes and MFC under low-velocity impact, considering strength and coupling effects of bending and stretching. Mansor et al. [42] investigated the energy absorption and impact properties of a tubular seamless type of FML fabricated from braided fiberglass sleeve and aluminum alloy tubes experimentally, and concluded that the mechanical properties of the FML tubes have good potential for application as an efficient energy absorber. Shiravand et al. [43] proposed an analytical solution for energy absorption of a conical FML tube consisting of any number of metal and laminate composite layers; the accuracy of analytical solutions was verified by experimental and numerical results.

As far as the authors know, little work has been carried out on theoretical research of the dynamic response of rectangular sandwich tubes with FML tubes and MFC subjected to low-velocity impacts. The main aim of this paper is to analytically and numerically investigate the low-velocity impacts on fully clamped slender rectangular sandwich tubes with FML tubes and a metal foam core. The organization of this article is as follows. The problem statement is presented in Section 2. In Section 3, an analytic solution is presented for predicting the dynamic response of a rectangular sandwich tube with FML tubes and MFC under low-velocity impacts. Section 4 presents the finite element analysis. Section 5 presents a comparison of the numerical and analytical results and discusses the influence of MVF, strength ratio factor of the two different material layers of the FML, and relative strength of the foam on the dynamic response of the rectangular sandwich tube. Section 6 contains concluding remarks.

## 2. Problem Formulation

**Figure 1:**
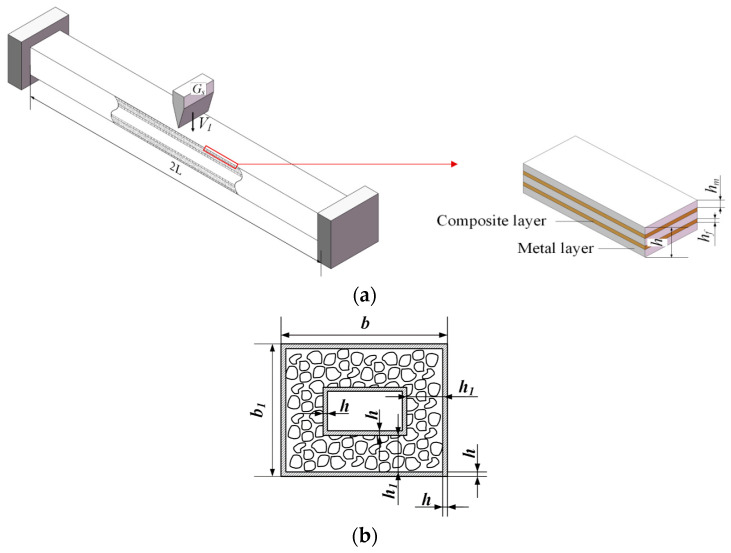
Sketch of a rectangular sandwich tube with FML tubes and MFC under heavy-impact with low-velocity at midspan. (**a**) Rectangular sandwich tube and (**b**) the cross-section of the rectangular sandwich tube. The red arrow indicates a partial enlarged view of the FML tube.

Now consider a fully clamped slender rectangular sandwich tube with FML tubes and a metal foam core, as shown in Figure 1. The tube length is 2*L* in length, and thelengths of cross-section are *b* in width and *b*_1_ in height. The mass per unit length of the rectangular sandwich tube is *G_b_*, and the FML tube is struck by a heavy impactor. The impactor has a mass *G_s_* and an initial low-velocity *V_I_*. Assume that the impactor is rigid. The MFC between the FML inner and outer tubes is assumed to be completely filled between them with no gaps, that is, it is perfectly bonded. As shown in Figure 1a,b, let the thickness of MFC be *h*_1_, and the thickness of FML be *h*, the thickness of the metal layer of FML be *h_m_*, and the thickness of the composite layer of FML be *h_f_*. In addition, the FML tube has *n* layers of metallic materials and *n* − 1 layers of composite materials in an alternating layup. Thus, the thickness of the FML tube is

(1) $$h=nh_{m}+(n-1)h_{f}$$

The density, strength, and elastic modulus of the composite layers and metal layers of FML tubes are *ρ_f_*, *ρ_m_*, *σ_f_*, *σ_m_*, *E_f_*, and *E_m_*, respectively. And the density, yield strength, and elastic modulus of the MFC are *ρ_c_*, *σ_c_*, and *E_c_*. Define a strength ratio factor of FML metal layer to FML composite layer *q*, so

(2) $$q=\frac{\sigma_{m}}{\sigma_{f}}$$

In order to better analyze the calculations, the different flow strengths and thicknesses of the components of the FML tubes are defined as the weighted strength *σ_fa_*. The weighted strength *σ_fa_* of the inner and outer rectangular tubes are supposed to follow the rigid–perfectly plastic law, while the yield strength *σ_c_* and densification strain *ε_D_* of the filled MFC are supposed to follow the rigid–perfectly plastic locking (*RPPL*) law.

## 3. Analytical Solutions

Rigid–plastic theory solutions can predict the dynamic behaviour of the FML when the plastic behaviour dominates the response of the plate [44]; the solution here is extended for predicting the dynamic response of rectangular sandwich tubes with FML tubes and MFC under low-velocity impact.

To describe the proportion of metal layers in FML, a metal volume fraction (MVF) *f* is defined, and can be given by

(3) $$f=\frac{nh_{m}}{h}\times 100\%$$

So, the weighted strength *σ_fa_* can be given by

(4) $$\sigma_{fa}=f\sigma_{m}+(1-f)\sigma_{f}$$

**Figure 2:**
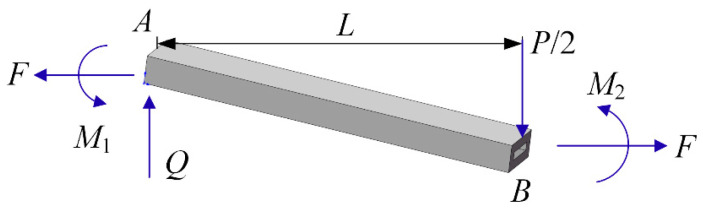
Global bending deformation model of the left plastic neutral surface of the rectangular sandwich tube with MFC under low-velocity impact at mid-span.

It is assumed that the ratio of height to span of the rectangular tube is small enough that the overall deformation of the rectangular tube does not produce a localized denting under the impactor. Therefore, the cross-section of the rectangular sandwich tube with FML tubes and MFC maintains its original shape, and the overall deformation profile under low-velocity impact is the same as that of a fully clamped whole rectangular tube. Therefore, the equation for the balance of the mass–tube system loaded at the mid-span of the rectangular sandwich tube can be derived as

(5) $$\left(\frac{G_{b}L^{2}}{3}+\frac{G_{s}L}{2}\right){\ddot{W}}_{0}+NW_{0}+2M_{1}=0$$

where

$$G_{b}=\left(4\rho_{f}h+2\rho_{c}h_{1}\right)\left(b+b_{1}-4h-2h_{1}\right),$$

in which *M*_1_ and *N* are the plastic moments and axial forces of the rectangular sandwich tube; the bending moment *M*_1_ = *M*_2_; at moderate deflections, *F* and *N* can be approximately equal; *W*_0_ is the deflection of the rectangular sandwich tube at impact point; ${\ddot{W}}_{0}$ is the acceleration of the impactor hitting the mid-span of the rectangular sandwich tube; *P* is the impact force of the impactor at impact position, as shown in Figure 2.

Zhang et al. [31] obtained the analytical solution of low-velocity impact analysis of a rectangular sandwich tube with MFC under low-velocity impact with a heavy-impactor. Appendix A shows the relationship between the dimensionless reaction force and the dimensionless deflection of the tube, and the relationship between the dimensionless kinetic energy of the striker and the maximum central deflection of a fully clamped rectangular sandwich tube with MFC subjected to low-velocity impact.

Inserting Equations (3) and (4) into Equations (A1) and (A3) yields the analytical solutions for the dynamic response of rectangular sandwich tubes with FML tubes and MFC subjected to low-velocity impact. Then, the relationship between the dimensionless initial impact energy $U_{K}^{*}$ and the dimensionless maximum central deflection $W_{0m}^{*}$, and the relationship between the dimensionless reaction force $P_{r}^{*}$ and the dimensionless deflection $W_{0}^{*}$ for the rectangular sandwich tube with FML tubes and MFC are given by

(6) $$\alpha U_{K}^{*}=\left\{\begin{array}{l} \frac{1}{6L_{2}}\left[2\left(n-1\right){\bar{h}}_{f}T+{\bar{h}}_{1}{\bar{\sigma }}_{c}p\left(1-f\right)\right]W_{0m}^{*}{\;}^{3}+W_{0m}^{*},\\ \;\;\;\;\;\;\;\;\;\;\;\;\;\;\;\;\;\;\;\;\;\;\;\;\;\;\;\;\;\;\;\;\;\;\;\;\;\;\;\;\;\;\;\;\;\;\;\;\;\;\;\;\;\;\;\;\;\;\;\;0\leq W_{0m}^{*}\leq \frac{\left(1-2{\bar{h}}_{1}p\right)\left(1-f\right)-4\left(n-1\right){\bar{h}}_{f}}{1-f}\\ \frac{W_{0m}^{*}{\;}^{3}}{12L_{2}}\left[2{\bar{h}}_{1}{\bar{\sigma }}_{c}p\left(1-f\right)+\left(1-2{\bar{h}}_{1}\right)p\left(1-f\right)T\right]+\frac{L_{5}}{4L_{2}}W_{0m}^{*}{\;}^{2}+\frac{L_{6}}{L_{2}}W_{0m}^{*}+S_{1}\\ \;\;\;\;\;\;\;\;\;\;\;\;\;\;\;+\frac{W_{0m}^{*}{\;}^{2}}{4L_{2}}\left[2{\bar{h}}_{1}{\bar{\sigma }}_{c}+\left(1-2{\bar{h}}_{1}\right)T\right]\left[3\left(n-1\right){\bar{h}}_{f}+\left(2{\bar{h}}_{1}p-1\right)\left(1-f\right)\right]p,\\ \;\;\;\;\;\;\;\;\;\;\;\;\frac{\left(1-2{\bar{h}}_{1}p\right)\left(1-f\right)-4\left(n-1\right){\bar{h}}_{f}}{1-f}\leq W_{0m}^{*}\leq \frac{\left(1-2{\bar{h}}_{1}p\right)\left(1-f\right)-2\left(n-1\right){\bar{h}}_{f}}{1-f}\\ -\frac{L_{4}}{12L_{2}}W_{0m}^{*}{\;}^{3}+\frac{1}{2}\left\{\frac{L_{4}}{2L_{2}}\left[\frac{\left(n-1\right){\bar{h}}_{f}}{1-f}+{\bar{h}}_{1}p-\frac{3}{2}\right]-\frac{L_{3}}{2L_{2}}\right\}W_{0m}^{*}{\;}^{2}+S_{2}\\ \;\;\;\;\;\;\;\;\;\;\;\;\;\;\;\;\;+\frac{W_{0m}^{*}}{L_{2}}\left(n-1\right){\bar{h}}_{f}\left[1-\frac{\left(n-1\right){\bar{h}}_{f}}{1-f}\right]T+\frac{L_{4}}{2L_{2}}\left\{{\left[\frac{\left(n-1\right){\bar{h}}_{f}}{1-f}\right]}^{2}-\frac{1}{4}\right\}W_{0m}^{*},\\ \;\;\;\;\;\;\;\;\;\;\;\;\;\;\;\;\;\;\;\;\;\;\;\;\;\;\;\;\;\frac{\left(1-2{\bar{h}}_{1}p\right)\left(1-f\right)-2\left(n-1\right){\bar{h}}_{f}}{1-f}\leq W_{0m}^{*}\leq \frac{1-f-2\left(n-1\right){\bar{h}}_{f}}{1-f}\\ \frac{W_{0m}^{*}{\;}^{3}}{12L_{2}}p\left(1-f\right)T+\frac{1}{2}\left[\frac{L_{1}}{L_{2}}-\frac{1}{2L_{2}}p\left(1-f\right)T\right]W_{0m}^{*}{\;}^{2}+\frac{W_{0m}^{*}}{4L_{2}}p\left(1-f\right)T+S_{3},\\ \;\;\;\;\;\;\;\;\;\;\;\;\;\;\;\;\;\;\;\;\;\;\;\;\;\;\;\;\;\;\;\;\;\;\;\;\;\;\;\;\;\;\;\;\;\;\;\;\;\;\;\;\;\;\;\;\;\;\;\;\;\;\;\;\;\;\;\;\;\;\;\;\;\;\;\;\;\;\;\;\;\;\frac{1-f-2\left(n-1\right){\bar{h}}_{f}}{1-f}\leq W_{0m}^{*}\leq 1\\ \frac{L_{1}}{2L_{2}}W_{0m}^{*}{\;}^{2}+S_{4},\;\;\;\;\;\;\;\;\;\;\;\;\;\;\;\;\;\;\;\;\;\;\;\;\;\;\;\;\;\;\;\;\;\;\;\;\;\;\;\;\;\;\;\;\;\;\;\;\;\;\;\;\;\;\;\;\;\;\;\;\;\;\;\;\;\;\;\;\;\;\;\;\;\;\;\;\;\;\;\;W_{0m}^{*}\geq 1 \end{array}\right.$$

and

(7) $$P_{r}^{*}=\left\{\begin{array}{l} \frac{3G^{*}}{3G^{*}+1}\left\{\frac{W_{0}^{*}{\;}^{2}}{2L_{2}}\left[2{\bar{h}}_{f}\left(n-1\right)T+{\bar{h}}_{1}{\bar{\sigma }}_{c}p\left(1-f\right)\right]+1\right\},\\ \;\;\;\;\;\;\;\;\;\;\;\;\;\;\;\;\;\;\;\;\;\;\;\;\;\;\;\;\;\;\;\;\;\;\;\;\;\;\;\;\;\;\;\;\;\;\;\;\;\;\;\;\;\;\;0\leq W_{0}^{*}\leq \frac{\left(1-2{\bar{h}}_{1}p\right)\left(1-f\right)-4\left(n-1\right){\bar{h}}_{f}}{1-f}\\ \frac{3G^{*}}{3G^{*}+1}\left\{p\left[2{\bar{h}}_{1}{\bar{\sigma }}_{c}+\left(1-2{\bar{h}}_{1}\right)T\right]\left[3{\bar{h}}_{f}\left(n-1\right)+\left(2{\bar{h}}_{1}p-1\right)\left(1-f\right)\right]\frac{W_{0}^{*}}{2L_{2}}\right\}\\ \;\;\;\;\;\;\;\;\;\;\;\;\;\;\;+\frac{3G^{*}}{3G^{*}+1}\left\{\frac{W_{0}^{*}{\;}^{2}}{4L_{2}}p\left(1-f\right)\left[2{\bar{h}}_{1}{\bar{\sigma }}_{c}+\left(1-2{\bar{h}}_{1}\right)T\right]+\frac{L_{5}}{2L_{2}}W_{0}^{*}+\frac{L_{6}}{L_{2}}\right\},\\ \;\;\;\;\;\;\;\;\;\;\;\;\;\frac{\left(1-2{\bar{h}}_{1}p\right)\left(1-f\right)-4\left(n-1\right){\bar{h}}_{f}}{1-f}\leq W_{0}^{*}\leq \frac{\left(1-2{\bar{h}}_{1}p\right)\left(1-f\right)-2\left(n-1\right){\bar{h}}_{f}}{1-f}\\ \frac{3G^{*}}{3G^{*}+1}\left\{\frac{1}{L_{2}}{\bar{h}}_{f}\left(n-1\right)pT\left[1-\frac{\left(n-1\right){\bar{h}}_{f}}{1-f}\right]+\frac{L_{4}}{2L_{2}}{\left[\frac{\left(n-1\right){\bar{h}}_{f}}{1-f}-\frac{1}{2}\right]}^{2}\right\}\\ \;\;\;\;\;\;\;\;\;\;\;\;+\frac{3G^{*}}{3G^{*}+1}\left\{\frac{L_{4}W_{0}^{*}{\;}^{2}}{8L_{2}}+\left[\frac{L_{4}}{2L_{2}}\left(\frac{n-1}{1-f}{\bar{h}}_{f}+{\bar{h}}_{1}p-\frac{1}{2}\right)-\frac{L_{3}}{2L_{2}}\right]W_{0}^{*}\right\},\\ \;\;\;\;\;\;\;\;\;\;\;\;\;\;\;\;\;\;\;\;\;\;\;\;\;\;\;\frac{\left(1-2{\bar{h}}_{1}p\right)\left(1-f\right)-2\left(n-1\right){\bar{h}}_{f}}{1-f}\leq W_{0}^{*}\leq \frac{1-f-2\left(n-1\right){\bar{h}}_{f}}{1-f}\\ \frac{3G^{*}}{3G^{*}+1}\left\{\frac{W_{0}^{*}{\;}^{2}+1}{4L_{2}}p\left(1-f\right)T+\left[\frac{L_{1}}{L_{2}}-\frac{1}{2L_{2}}p\left(1-f\right)T\right]W_{0}^{*}\right\},\\ \;\;\;\;\;\;\;\;\;\;\;\;\;\;\;\;\;\;\;\;\;\;\;\;\;\;\;\;\;\;\;\;\;\;\;\;\;\;\;\;\;\;\;\;\;\;\;\;\;\;\;\;\;\;\;\;\;\;\frac{\left(1-2{\bar{h}}_{1}p\right)\left(1-f\right)-2\left(n-1\right){\bar{h}}_{f}}{1-f}\leq W_{0}^{*}\leq 1\\ \frac{3G^{*}}{3G^{*}+1}\frac{L_{1}}{L_{2}}W_{0}^{*},\;\;\;\;\;\;\;\;\;\;\;\;\;\;\;\;\;\;\;\;\;\;\;\;\;\;\;\;\;\;\;\;\;\;\;\;\;\;\;\;\;\;\;\;\;\;\;\;\;\;\;\;\;\;\;\;\;\;\;\;\;\;\;\;\;\;\;\;\;\;\;\;\;\;\;\;W_{0}^{*}\geq 1 \end{array}\right.$$

where

$$\begin{array}{l} {\bar{h}}_{f}=\frac{h_{f}}{b_{1}},\;{\bar{\rho }}_{c}=\frac{\rho_{c}}{\rho_{f}},\;{\bar{\sigma }}_{c}=\frac{\sigma_{c}}{\sigma_{f}},\;q=\frac{\sigma_{m}}{\sigma_{f}},\;T=\left(q-1\right)f+1,\;\bar{V}=\frac{V_{0}}{\sqrt{\sigma_{f}/\rho_{f}}},\;\bar{t}=\frac{t}{b_{1}\sqrt{\rho_{f}/\sigma_{f}}},\\ L_{1}=2{\bar{h}}_{f}p\left(n-1\right)\left({\bar{h}}_{1}{\bar{\sigma }}_{c}+T\right)+{\bar{h}}_{1}{\bar{\sigma }}_{c}p\left[p\left(1-f\right)-2{\bar{h}}_{f}\left(n-1\right)\right]\\ \;\;-\left[{\bar{h}}_{1}{\bar{\sigma }}_{c}p\left(1-f\right)+2{\bar{h}}_{f}\left(n-1\right)T\right]\left[\frac{4\left(n-1\right){\bar{h}}_{f}}{1-f}+2{\bar{h}}_{1}p-1\right],\\ L_{2}={\bar{h}}_{1}p\left[1-\frac{2\left(n-1\right){\bar{h}}_{f}}{1-f}-{\bar{h}}_{1}p\right]\left\{2{\bar{h}}_{f}\left(n-1\right)T+\left[p\left(1-f\right)-2{\bar{h}}_{f}\left(n-1\right)\right]{\bar{\sigma }}_{c}\right\}\\ \;\;+\left[\left(1-2{\bar{h}}_{1}\right)T+2{\bar{h}}_{1}{\bar{\sigma }}_{c}\right]\left[1-f-3{\bar{h}}_{f}\left(n-1\right)-2{\bar{h}}_{1}p\left(1-f\right)\right]p\frac{\left(n-1\right){\bar{h}}_{f}}{1-f}\\ \;\;+\frac{1}{2}\left[2{\bar{h}}_{f}\left(n-1\right)T+p\left(1-f\right){\bar{h}}_{1}{\bar{\sigma }}_{c}\right]{\left[1-\frac{4\left(n-1\right){\bar{h}}_{f}}{1-f}-2{\bar{h}}_{1}p\right]}^{2}\\ \;\;+{\bar{h}}_{f}pT\left(n-1\right)\left[1-\frac{\left(n-1\right){\bar{h}}_{f}}{1-f}\right],\\ L_{3}=-2{\bar{h}}_{f}p\left(n-1\right)\left[2{\bar{h}}_{1}{\bar{\sigma }}_{c}+\left(1-2{\bar{h}}_{1}\right)T\right]\\ \;\;-\left[4{\bar{h}}_{f}\left(n-1\right)T+2{\bar{h}}_{1}{\bar{\sigma }}_{c}p\left(1-f\right)\right]\left[1-\frac{4\left(n-1\right){\bar{h}}_{f}}{1-f}-2{\bar{h}}_{1}p\right],\\ L_{4}=4{\bar{h}}_{f}\left(n-1\right)T+2{\bar{\sigma }}_{c}\left[p\left(1-f\right)-2{\bar{h}}_{f}\left(n-1\right)\right],\\ L_{5}=p{\bar{h}}_{f}\left(n-1\right)\left[2{\bar{h}}_{1}{\bar{\sigma }}_{c}+\left(1-2{\bar{h}}_{1}\right)T\right]\\ \;\;+\left[2{\bar{h}}_{1}{\bar{\sigma }}_{c}p\left(1-f\right)+4{\bar{h}}_{f}\left(n-1\right)T\right]\left[1-\frac{4\left(n-1\right){\bar{h}}_{f}}{1-f}-2{\bar{h}}_{1}p\right],\\ L_{6}={\bar{h}}_{f}pT\left(n-1\right)\left[1-\frac{\left(n-1\right){\bar{h}}_{f}}{1-f}\right]+\frac{p}{4}\left(1-f\right)\left[\left(1-2{\bar{h}}_{1}\right)T+2{\bar{h}}_{1}{\bar{\sigma }}_{c}\right]{\left[1-\frac{2\left(n-1\right){\bar{h}}_{f}}{1-f}-2{\bar{h}}_{1}p\right]}^{2}\\ +{\bar{h}}_{1}p\left[1-\frac{2\left(n-1\right){\bar{h}}_{f}}{1-f}-{\bar{h}}_{1}p\right]\left\{\left[2{\bar{h}}_{f}\left(n-1\right)T\right]+\left[p\left(1-f\right)-2{\bar{h}}_{f}\left(n-1\right)\right]{\bar{\sigma }}_{c}\right\},\\ S_{1}=\left(1-\frac{L_{6}}{L_{2}}\right)\left[1-\frac{4\left(n-1\right){\bar{h}}_{f}}{1-f}-2{\bar{h}}_{1}p\right]-\frac{L_{5}}{4L_{2}}{\left[1-\frac{4\left(n-1\right){\bar{h}}_{f}}{1-f}-2{\bar{h}}_{1}p\right]}^{2}\\ \;\;+\frac{T}{6L_{2}}\left[2{\bar{h}}_{f}\left(n-1\right)+p\left(1-f\right)\left({\bar{h}}_{1}-\frac{1}{2}\right)\right]{\left[1-\frac{4\left(n-1\right){\bar{h}}_{f}}{1-f}-2{\bar{h}}_{1}p\right]}^{3}\\ \;\;-\frac{p}{4L_{2}}\left[3{\bar{h}}_{f}\left(n-1\right)+\left(2{\bar{h}}_{1}p-1\right)\left(1-f\right)\right]\left[2{\bar{h}}_{1}{\bar{\sigma }}_{c}+\left(1-2{\bar{h}}_{1}\right)T\right]{\left[1-\frac{4\left(n-1\right){\bar{h}}_{f}}{1-f}-2{\bar{h}}_{1}p\right]}^{2},\\ S_{2}=\frac{1}{24L_{2}}\left[4{\bar{h}}_{1}p\left(1-f\right){\bar{\sigma }}_{c}-4{\bar{h}}_{1}Tp\left(1-f\right)-L_{4}+2p\left(1-f\right)T\right]{\left[1-\frac{2\left(n-1\right){\bar{h}}_{f}}{1-f}-2{\bar{h}}_{1}p\right]}^{3}\\ \;\;\;\;\;+\frac{p}{4L_{2}}\left[3{\bar{h}}_{f}\left(n-1\right)+\left(2{\bar{h}}_{1}p-1\right)\left(1-f\right)\right]\left[2{\bar{h}}_{1}{\bar{\sigma }}_{c}+\left(1-2{\bar{h}}_{1}\right)T\right]{\left[1-\frac{2\left(n-1\right){\bar{h}}_{f}}{1-f}-2{\bar{h}}_{1}p\right]}^{2}\\ \;\;\;\;\;-\frac{1}{4L_{2}}\left\{\left[L_{4}\left(\frac{n-1}{1-f}{\bar{h}}_{f}+{\bar{h}}_{1}p-\frac{1}{2}\right)-L_{3}-L_{5}\right]{\left[1-\frac{2\left(n-1\right){\bar{h}}_{f}}{1-f}-2{\bar{h}}_{1}p\right]}^{2}\right\}+S_{1}\\ \;\;\;\;\;+\frac{1}{L_{2}}\left[1-\frac{2\left(n-1\right){\bar{h}}_{f}}{1-f}-2{\bar{h}}_{1}p\right]\left\{L_{6}-{\bar{h}}_{f}pT\left(n-1\right)\left[1-\frac{\left(n-1\right){\bar{h}}_{f}}{1-f}\right]-\frac{L_{4}}{2}{\left[\frac{\left(n-1\right){\bar{h}}_{f}}{1-f}-\frac{1}{2}\right]}^{2}\right\},\\ S_{3}=-\frac{1}{12L_{2}}\left[L_{4}+p\left(1-f\right)T\right]{\left[1-\frac{2\left(n-1\right){\bar{h}}_{f}}{1-f}\right]}^{3}\\ \;\;+\frac{1}{4L_{2}}\left[L_{4}\left(\frac{n-1}{1-f}{\bar{h}}_{f}+{\bar{h}}_{1}p-\frac{3}{2}\right)-L_{3}-2L_{1}+p\left(1-f\right)T\right]{\left[1-\frac{2\left(n-1\right){\bar{h}}_{f}}{1-f}\right]}^{2}\\ \;\;+\frac{1}{L_{2}}\left\{{\bar{h}}_{f}pT\left(n-1\right)\left[1-\frac{\left(n-1\right){\bar{h}}_{f}}{1-f}\right]\left[1-\frac{2\left(n-1\right){\bar{h}}_{f}}{1-f}\right]\right\}\\ \;\;+\frac{L_{4}}{2L_{2}}\left[{\left(\frac{n-1}{1-f}{\bar{h}}_{f}\right)}^{2}-\frac{1}{4}\right]\left[1-\frac{2\left(n-1\right){\bar{h}}_{f}}{1-f}\right]-\frac{pT}{4L_{2}}\left(1-f\right)\left[1-\frac{2\left(n-1\right){\bar{h}}_{f}}{1-f}\right]+S_{2},\\ S_{4}=\frac{1}{12L_{2}}pT\left(1-f\right)+S_{3}. \end{array}$$

## 4. Finite Element Analysis

This section presents a numerical study of low-velocity impact of a clamped rectangular sandwich tube with FML tubes and MFC using ABAQUS/Explicit software. In this model, both the FML tubes and the MFC are modeled with 3D eight-node linear hexahedral brick elements (type C3D8R). A rigid roller is modeled to simulate the impactor, defining a point at its center as a point of concentrated mass and predetermining an initial low-velocity. The degrees of freedom of the nodes at both ends of the rectangular sandwich tube are set to zero to simulate a fixed support, that is, all vertical, horizontal, and rotational displacements are zero. In addition, the contact surface of the rigid roller with the outer face of the FML outer tube is set to be frictionless contact.

Take the half-span length of the rectangular sandwich tube as *L* = 200 mm, and the width and height of the cross-section of the outer FML tube are *b* = 40 mm and *b*_1_ = 16 mm. The thickness of the metal foam is *h*_1_ = 3 mm, and the thicknesses of the metal layer and the composite layer of the FML tube are *h_m_* = 0.2 mm and *h_f_* = 0.1 mm, respectively. For the convenience of this study, there are three metal layers, that is, *n* = 3, and then there are two composite layers. Thus, the total thickness of the FML tube is *h* = 0.8 mm, that is, $\bar{h}$ = 4/15, ${\bar{h}}_{1}$ = 0.075, ${\bar{h}}_{f}$ = 1/160, *p* = 2.5, and *f* = 0.75, respectively. In addition, a suitable radius is chosen for the loading roller of *R* = 4.

Based on experimental data [45], the metal layer of the FML is made of aluminum alloy, and the glass composite woven layer includes quasi-isotropic glass fiber fabrics infused with vinylester resin. The composite layers of the FML tube are made of composite material with strength *σ_f_* = 220 MPa, elastic modulus *E_f_* = 10 Gpa, elastic Poisson’s ratio *ν_ef_* = 0.3, and density *ρ_f_* = 1700 kg/m^3^, respectively. Due to the stretchable nature of FML tubes, composite layers can be assumed to be linearly elastic [45]. Using aluminum as the metal layers of the FML tubes, the yield strength, elastic modulus, elastic Poisson’s ratio, density, and linear hardening tangential modulus are *σ_m_* = 460 Mpa, *E_m_* = 70 Gpa, *ν_em_* = 0.3, *ρ_m_* = 2800 kg/m^3^, and *E_mt_* = 0.02*E_m_* respectively. It is assumed that the layers of the metal are able to withstand deformation without fracture, that is, they have sufficient ductility.

The plastic compressible behavior of MFC Is modeled In ABAQUS software using the Deshpande–Fleck constitutive model [46]. The yield strength, elastic modulus, elastic Poisson’s ratio, plastic Poisson’s ratio, and density are *σ_c_* = 10 Mpa, *E_c_* = 10 Gpa, *ν_ec_* = 0.3, *ν_p_* = 0, and *ρ_c_* = 405 kg/m^3^, respectively, for aluminum foam as an isotropic metallic foam core. The MFC has a long yield stress platform *σ_c_* that continues up to the densification strain *ε_D_* = 0.5, and it is assumed that the MFC obeys a linear hardening law with tangent modulus *E_ct_* = 0.3*E_m_* beyond densification.

**Figure 3:**
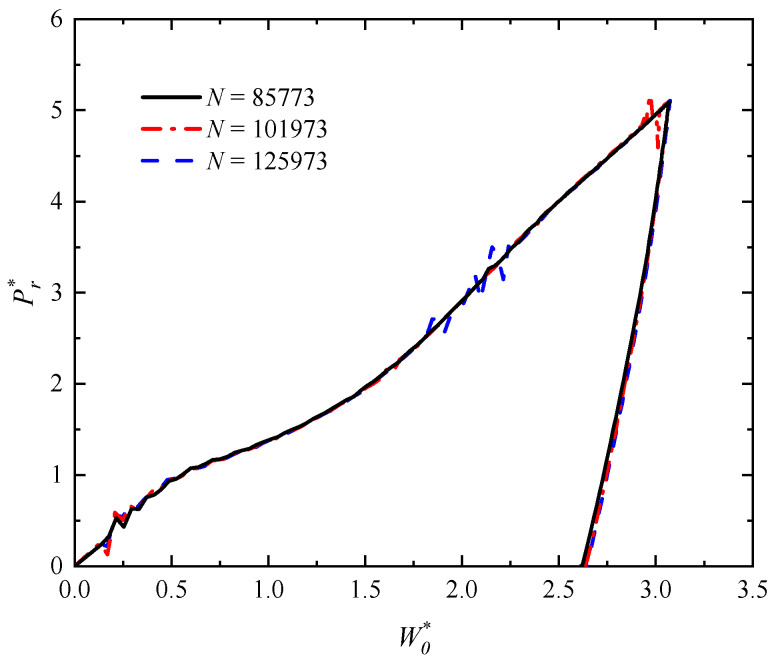
Results of the mesh sensitivity examination of the relationship between impact force and deflection for the rectangular sandwich tube with FML tubes and MFC under low-velocity impact with G* = 1000 and *V_I_* = 2.5 m/s.

A calculation of mesh sensitivity is performed to ensure that the mesh division does not have an impact on the results. Figure 3 shows the results of the mesh sensitivity examination of impact force versus deflection of outer surface of the rectangular sandwich tube with FML tubes and MFC under low-velocity impact at mid-span with G* = 1000 and *V_I_* = 2.5 m/s. In this model, the number of elements is *N* = 85,773. It can be seen that extra mesh refinement does not significantly change the results of the calculations.

## 5. Results and Discussion

**Figure 4:**
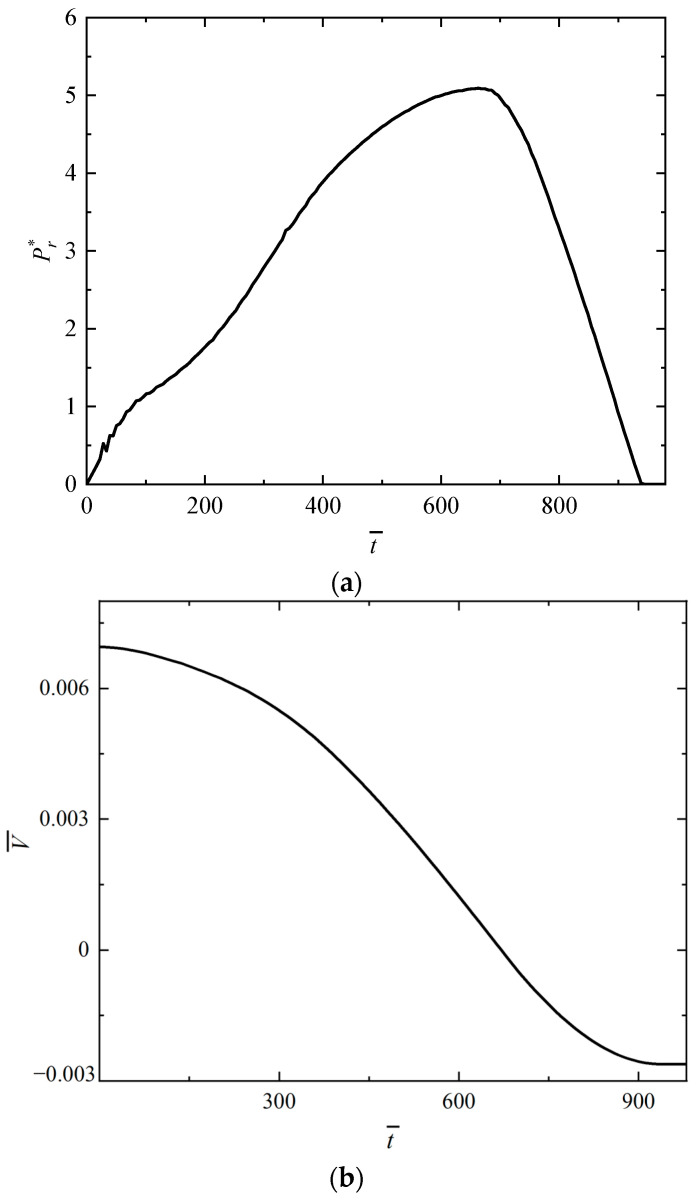
Numerical results of the impact response for the rectangular sandwich tube with FML tubes and MFC under low-velocity impact with G* = 1000, *V_I_* = 2.5 m/s. (**a**) The relationship between the impact force of the impactor and time, and (**b**) the relationship between the velocity of the impactor and time.

The impact force and velocity of the impactor versus time curves are shown in Figure 4a,b. It can be seen in Figure 4a that the impact force of the impactor gradually increases to the maximum value and then decreases to zero during the impact process, indicating that the impactor causes the FML rectangular sandwich tube to deflect maximally and then is rebounded until it leaves the surface of the FML rectangular sandwich tube. As can be seen in Figure 4b, the velocity of the impactor gradually decreases to zero and then increases in the opposite direction, indicating that the impactor rebounds after impact.

**Figure 5:**
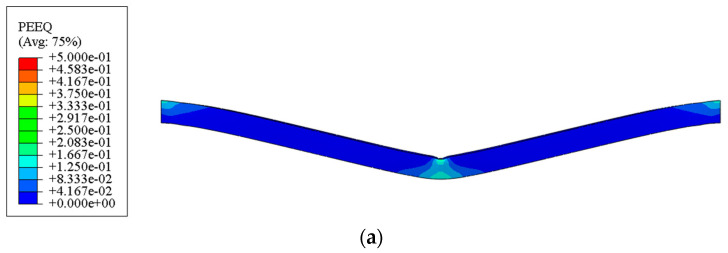

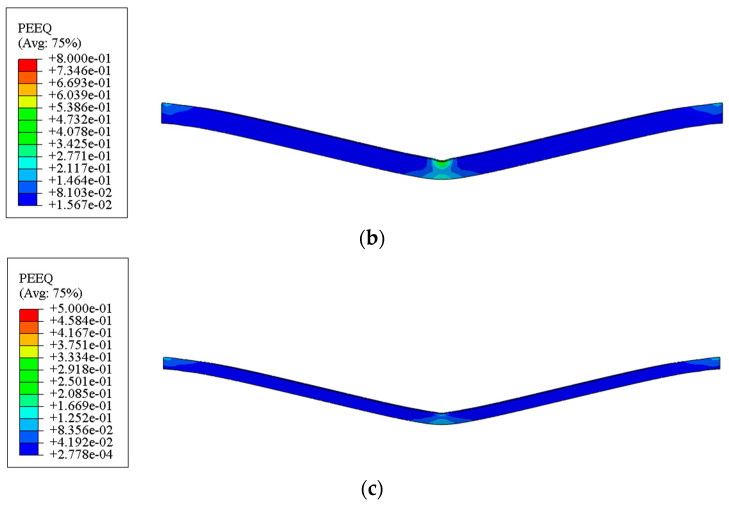
Numerical results of the equivalent plastic strain distribution of rectangular sandwich tubes with FML tubes and MFC impacted by a heavy mass of G* = 1000 and an initial low-velocity of *V_I_* = 2.5 m/s. (**a**) The outer tube of the rectangular sandwich tube, (**b**) the MFC of the rectangular sandwich tube, and (**c**) the inner tube of the rectangular sandwich tube. It should be noted that “5e-01” means “5 × 10^−1^” in the color bars.

Figure 5a–c show the distribution of equivalent plastic strain (PEEQ) for the outer FML tube, MFC, and inner FML tube of the rectangular sandwich tubes. It can be seen that the local denting of the tube is not obvious below the impactor.

**Figure 6:**
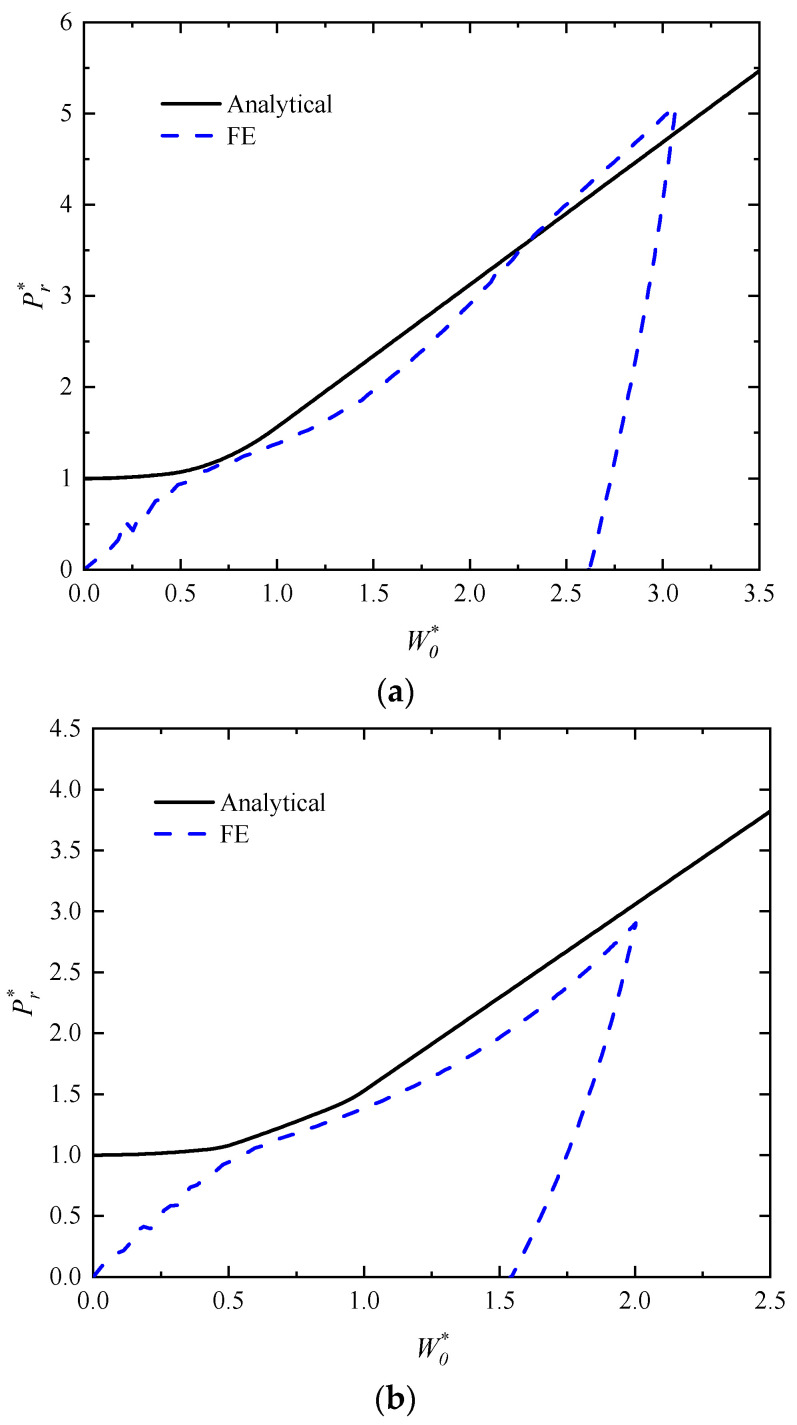
Analytical predictions and numerical results of the relationship between the dimensionless impact force and deflection for the rectangular sandwich tubes with FML tubes and MFC under low-velocity impact with a heavy mass impactor. (**a**) G* = 1000, *V_I_* = 2.5 m/s and (**b**) G* = 2500, *V_I_* = 1 m/s.

The analytical predictions and numerical results of the relationship between the dimensionless impact force $P_{r}^{*}$ and dimensionless deflection $W_{0}^{*}$ for the rectangular sandwich tubes with FML tubes and MFC under low-velocity heavy-mass impact at mid-span are shown in Figure 6a,b. The geometrical and material parameters are *f* = 0.75, *p* = 2.5, *q* = 23/11, $\bar{h}$ = 4/15, ${\bar{h}}_{1}$ = 0.075, and ${\bar{\sigma }}_{c}$ = 1/22. It is known from the figure that the curve has an upward trend and the impact force increases with the increase in deflection in analytical predictions. It can also be seen that the results obtained from the analytical predictions are reasonably well consistent with the numerical results of the post-yield phase. However, there are some differences between the analytical predictions and numerical results, which is probably caused by the effects of the shear force, elastic deformation, inertia, and strain hardening of the material that are neglected in the analytical solutions. It can be noticed that the numerical results show micro-oscillations in the initial stage, which are most likely due to the complex interaction between the impactor and the rectangular sandwich tubes.

**Figure 7:**
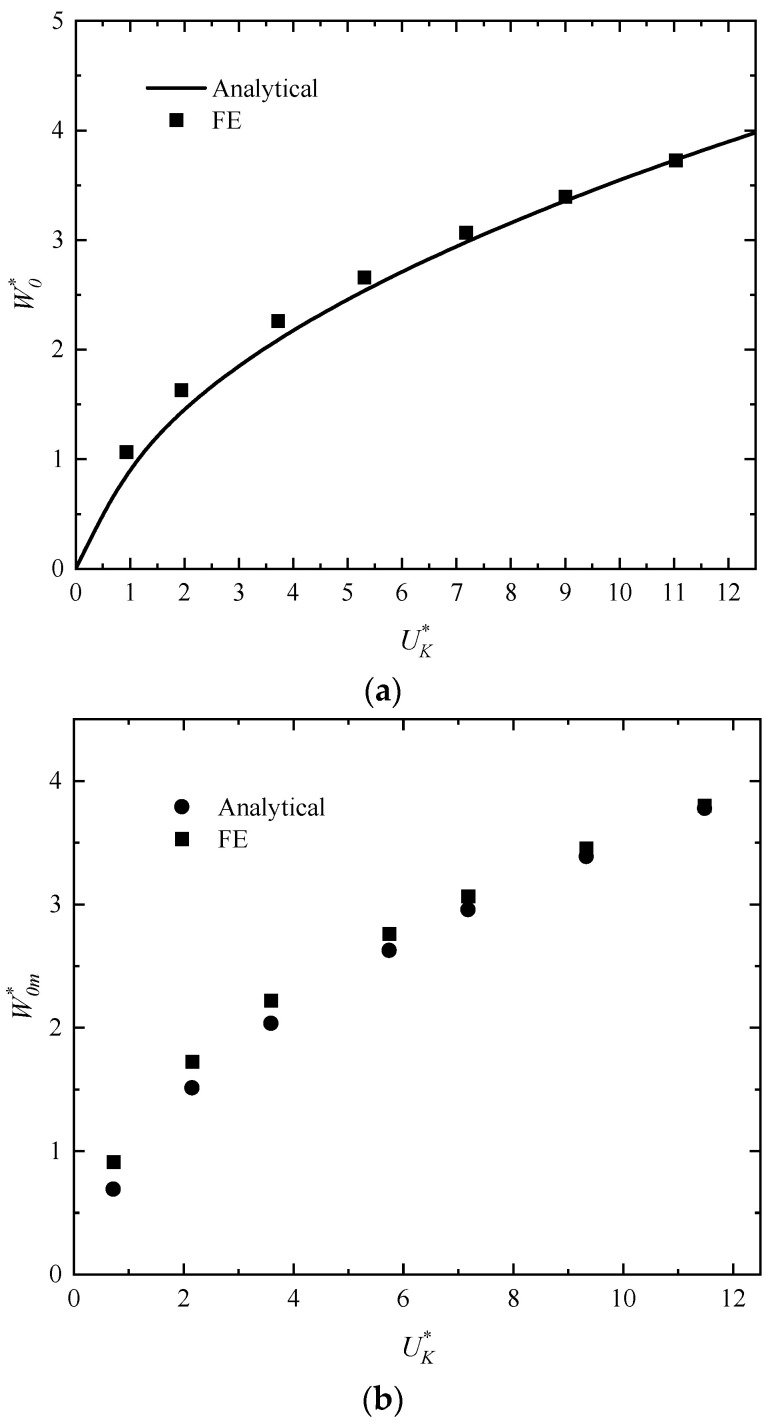
Comparisons of analytical and numerical results of the dimensionless maximum deflection for the middle span of the outer surface of the rectangular sandwich tubes with FML tubes and MFC versus the initial impact energy of the impactor with (**a**) a given constant mass ratio G* = 1000 and (**b**) a given constant initial velocity *V_I_* = 2.5 m/s.

Figure 7a,b show the analytical results and numerical results of the dimensionless maximum deflection $W_{0m}^{*}$ for the middle span of the outer surface of the rectangular sandwich tubes versus initial impact energy $U_{K}^{*}$ of the impactor, where *f* = 0.75, *p* = 2.5, *q* = 23/11, $\bar{h}$ = 4/15, ${\bar{h}}_{1}$ = 0.075, and ${\bar{\sigma }}_{c}$ = 1/22. It is known from the figure that the curve has an upward trend and the maximum deflection increases with the increase ininitial impact energy in analytical predictions. In Figure 7a, it can be seen that the maximum deflection increases with the increase in impact energy when the mass ratio is constant as G* = 1000. The cases for given initial velocity *V_I_* = 2.5 m/s and various mass ratio G* are considered in Figure 7b. It can be seen that the analytical predictions are in good harmony with the numerical results, while the numerical results may slightly overestimate the analytical predictions. The disagreement between the analytical predictions and numerical results is probably caused by ignoring the influence of elastic deformation and strain hardening of material in the analytical solution.

Based on the analytical solutions, two types of curves are analyzed here, i.e., the dimensionless impact force $P_{r}^{*}$ versus deflection $W_{0}^{*}$ for rectangular sandwich tubes with FML tubes and MFC, and the dimensionless maximum deflection $W_{0m}^{*}$ for the middle span of the outer surface versus initial impact energy $U_{K}^{*}$ of the impactor of the rectangular sandwich tubes. The discussion here focuses on the influence of the MVF *f*, the strength ratio factor *q*, and the foam strength ${\bar{\sigma }}_{c}$ in the FML tubes on these two curves.

**Figure 8:**
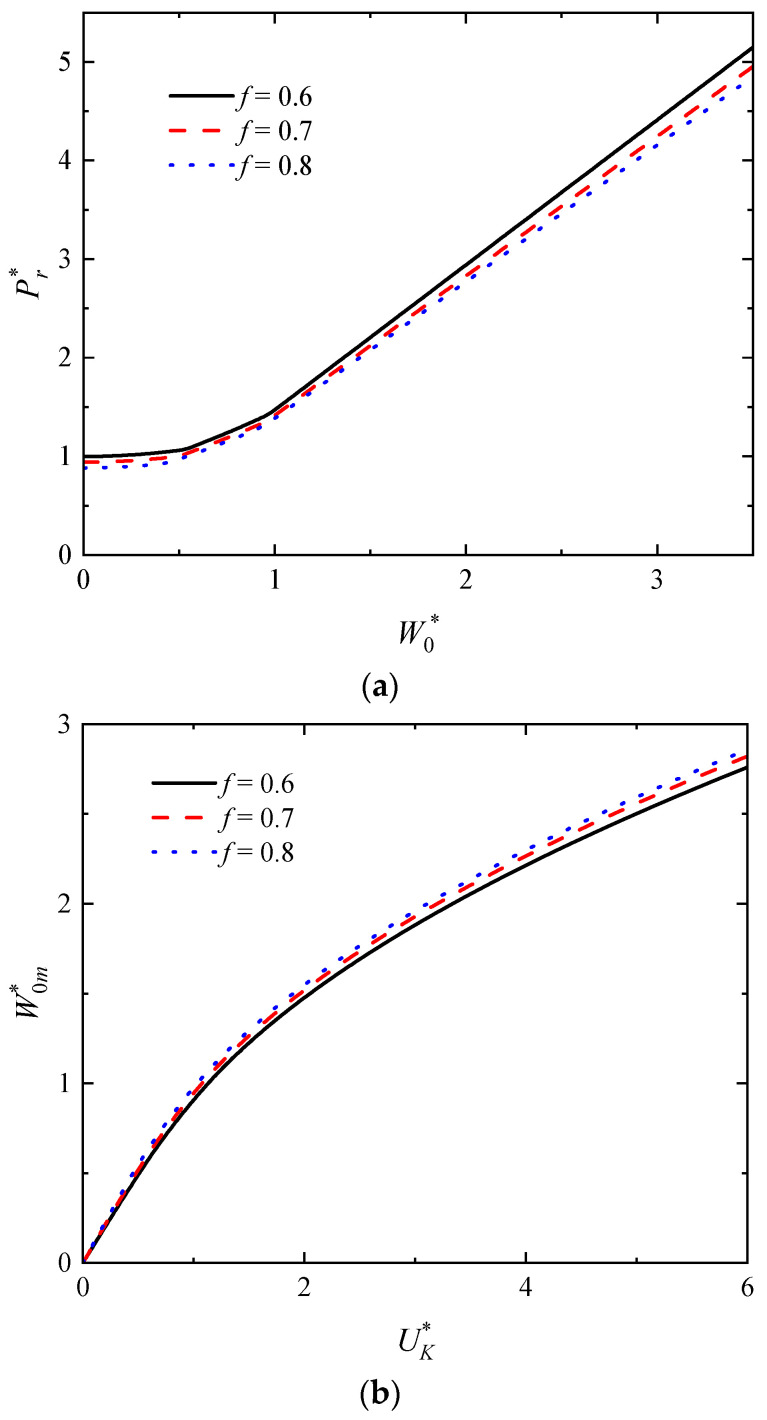
The influence of the MVF *f* on the dynamic response of rectangular sandwich tubes with FML tubes and MFC under low-velocity impact. (**a**) The curves of $W_{0}^{*}$ versus $P_{r}^{*}$ and (**b**) the curves of $U_{K}^{*}$ versus $W_{0m}^{*}$.

The Influence of the MVF *f* on the $W_{0}^{*}$ versus $P_{r}^{*}$ curves as well as the $U_{K}^{*}$ versus $W_{0m}^{*}$ curves of the rectangular sandwich tubes with FML tubes are shown in Figure 8a,b, respectively, in which *n* = 3, *p* = 2.5, *q* = 23/41, $\bar{h}$ = 4/15, ${\bar{h}}_{1}$ = 0.075, ${\bar{\sigma }}_{c}$ = 1/82, G* = 1000. The smaller the MVF *f*, the greater the fiber content in the FML. As shown in Figure 8a, the impact force increases when the MVF *f* decreases for the constant deflection, indicating that the greater the fiber content in the FML, the better the load-bearing capacity of the rectangular sandwich tube. In Figure 8b, when the initial impact energy is constant, the maximum deflection of the rectangular sandwich tube decreases with decreasing MVF *f*, indicating that the greater the fiber content in the FML, the better the impact resistance of the rectangular sandwich tube.

**Figure 9:**
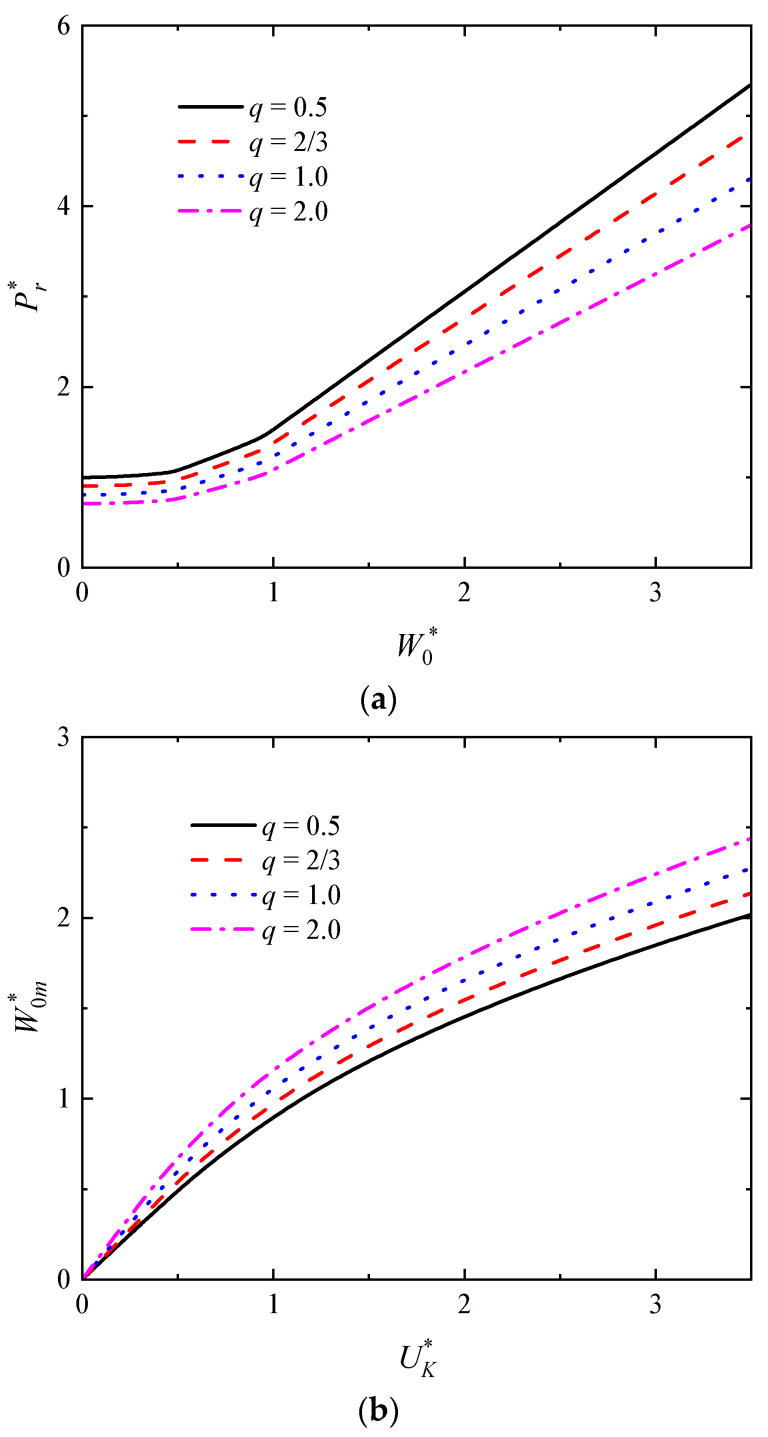
The influence of the strength ratio *q* on the dynamic response of rectangular sandwich tubes with FML tubes and MFC under low-velocity impact. (**a**) The curves of $W_{0}^{*}$ versus $P_{r}^{*}$ and (**b**) the curves of $U_{K}^{*}$ versus $W_{0m}^{*}$.

The influence of the strength ratio factor *q* of the FML metal layer to FML composite layer on the $W_{0}^{*}$ versus $P_{r}^{*}$ curve as well as the $U_{K}^{*}$ versus $W_{0m}^{*}$ curves of rectangular sandwich tubes with FML tubes are shown in Figure 9a,b, respectively, in which *n* = 3, *p* = 2.5, *f =* 0.75, $\bar{h}$ = 4/15, ${\bar{h}}_{1}$ = 0.075, G* = 1000. When the strength of the metal layer in the FML is constant, as the strength of the fiber layer in the FML increases, the value of *q* decreases. In Figure 9a, the impact force increases obviously with the decreasing strength ratio when the deflection is constant, indicating that the higher the strength of the fiber in the FML, the better the load-carrying capacity of the rectangular sandwich tube. Also, the dimensionless maximum deflection of the rectangular sandwich tube decreases with the decreasing strength ratio for a constant initial impact energy, as shown in Figure 9b, indicating that the greater the strength of the fiber in the FML, the better the impact resistance of the rectangular sandwich tube. In addition, it is shown that the effect of *q* on both the load-carrying capacity and the impact resistance of the rectangular sandwich tube is evident. It can be seen that the strength ratio plays an important role in the low-velocity impact of the rectangular sandwich tube with FML tubes.

**Figure 10:**
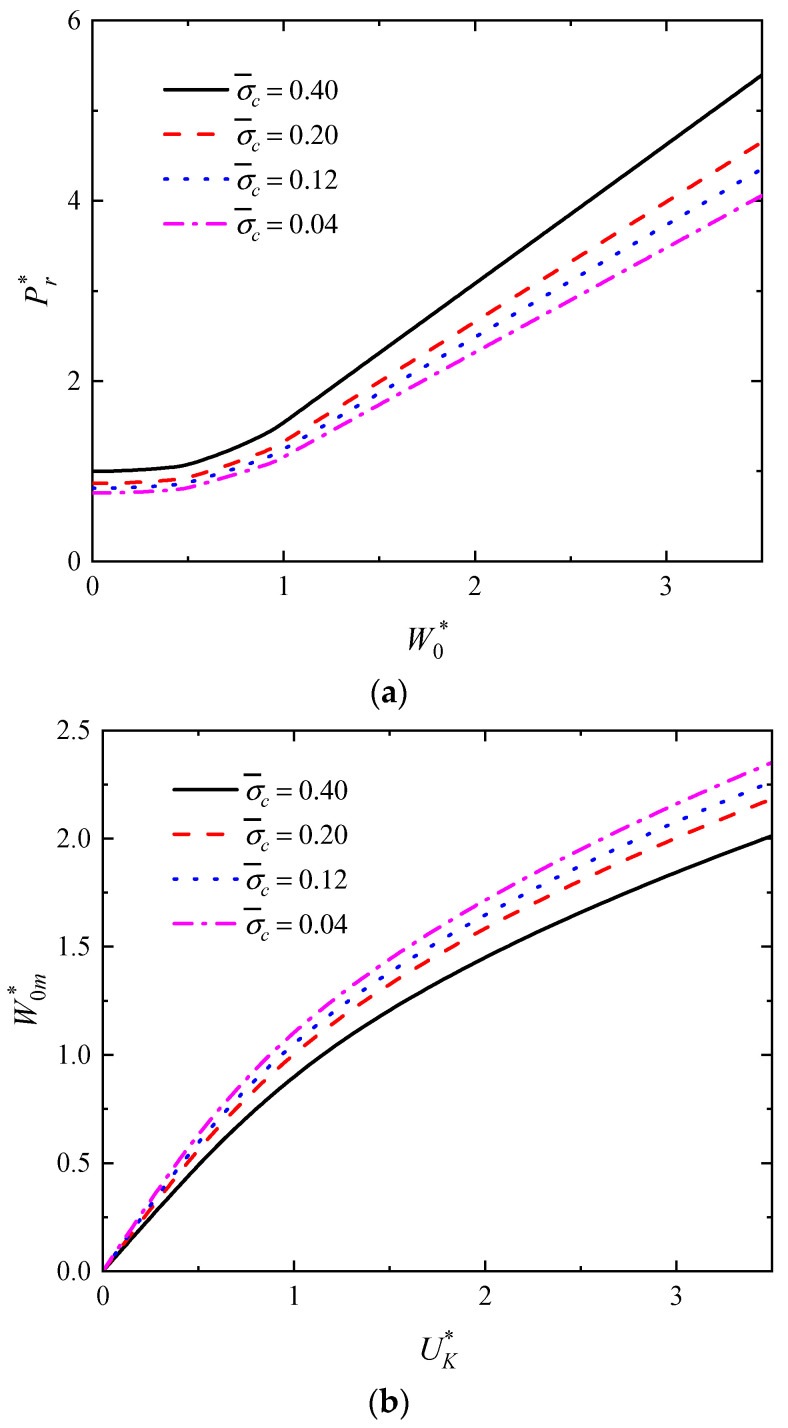
The influence of the foam strength ${\bar{\sigma }}_{c}$ on the dynamic response of rectangular sandwich tubes with FML tubes and MFC under low-velocity impact. (**a**) The curves of $W_{0}^{*}$ versus $P_{r}^{*}$ and (**b**) the curves of $U_{K}^{*}$ versus $W_{0m}^{*}$.

In addition, the influence of the foam strength ${\bar{\sigma }}_{c}$ on the $W_{0}^{*}$ versus $P_{r}^{*}$ curves as well as the $U_{K}^{*}$ versus $W_{0m}^{*}$ curves are investigated as shown in Figure 10a,b, respectively, where *n* = 3, *p* = 2.5, *f =* 0.75, *q* = 23/11, $\bar{h}$ = 4/15, ${\bar{h}}_{1}$ = 0.075, G* = 1000. As shown in Figure 10a, the impact force increases with increasing foam strength when the deflection is constant. Moreover, as shown in Figure 10b, the dimensionless maximum deflection of the rectangular sandwich tube decreases with increasing foam strength for a constant initial impact energy. It can be concluded that the higher the strength of the foam, the higher the load-carrying capacity and the better the impact resistance of the rectangular sandwich tube.

## 6. Concluding Remarks

In this paper, the dynamic response of clamped rectangular sandwich tubes with FML tubes and MFC subjected to the impact of a low-velocity and heavy-mass impactor is theoretically and numerically investigated. As a new type of composite tube combining polymer and metal materials, FML tube plays a good role in the energy absorption and dynamic response of foam-filled tubes under low-velocity impacts. Based on modified rigid–plastic material approximation and introducing the number and volume fraction of composite layup, simple formulations for low-velocity impacts of rectangular sandwich tubes with FML tubes and MFC are obtained. The analytical solution agrees with the numerical results reasonably well. It is shown that the impact force of the rectangular sandwich tubes with FML tubes and MFC increases with increasing MVF, strength ratio factor of the FML metal layer to the FML composite layer, and relative strength of the foam. Moreover, the maximum deflection of the rectangular sandwich tubes with FML tubes and MFC decreases with increasing MVF, strength ratio factor, and foam strength. It is shown that by increasing the fiber content and fiber strength of the FML tubes and the foam strength, the load-carrying and energy-absorbing capacity of the rectangular sandwich tubes can be effectively improved, especially by changing the fiber properties. In addition, the present analytical model can be reasonably applied to make predictions about the dynamic response of rectangular sandwich tubes with FML tubes and MFC under low-velocity impacts. The analytical model also provides a theoretical basis for the design of FML tubes.

## Data Availability

The authors attest that all data for this study are included in the paper.

## Appendix A. Analytical Solutions of a Fully Clamped Rectangular Sandwich Metal Tube Subjected to Low-Velocity Impact

This appendix contains the relationship between the dimensionless reaction force and the dimensionless deflection for a fully clamped rectangular sandwich metal tubewith MFC subjected to low-velocity impact, as well as the relationship between the dimensionless kinetic energy of the striker and maximum central deflection of the tube [31].

Rectangular sandwich tubes with MFC consist of an inner and outer rectangular tube of thickness *h* and a MFC of thickness *h*_1_, in which the width, height, and length of the rectangular sandwich tube are *b*, *b*_1_, 2*L* and the mass per unit length of the rectangular sandwich tube is *G_b_*, respectively. The density of the inner and outer rectangular tube is *ρ_f_*, and the density of isotropic MFC is *ρ_c_*. The rectangular sandwich tube with MFC is impacted by an impactor with a heavy mass *G_s_* and an initial velocity of *V_I_*. The inner and outer rectangular tube are supposed to follow the RPP law of yield strength *σ_f_*, and the filled MFC follows the RPPL material of yield strength *σ_c_* and densification strain *ε_D_*. Based on the flow rules associated with the fully clamped rectangular sandwich tubewith MFC at both ends, the analytical solution can be obtained using the yield condition [47] and the expressions for the elongation and angular rotation.

The relationship between the dimensionless kinetic energy of the initial impact $U_{K}^{*}$ and the dimensionless maximum central deflection $W_{0m}^{*}$ of the rectangular sandwich metal tube with MFC under low-velocity impact can be expressed as

(A1) $$\alpha U_{K}^{*}=\left\{\begin{array}{l} \frac{1}{6B}\left(\frac{2\bar{h}}{p}+{\bar{h}}_{1}\bar{\sigma }\right)W_{0m}^{*}{\;}^{3}+W_{0m}^{*},\;\;\;\;\;\;\;\;\;\;\;\;\;\;\;\;\;\;\;\;\;\;\;\;\;\;\;\;\;\;\;\;\;\;\;0\leq W_{0m}^{*}\leq 1-2{\bar{h}}_{1}p-4\bar{h}\\ \begin{array}{l} \frac{W_{0m}^{*}{\;}^{3}}{12B}\left(2{\bar{h}}_{1}\bar{\sigma }+1-2{\bar{h}}_{1}\right)+\frac{1}{4B}\left(3\bar{h}+2{\bar{h}}_{1}p-1\right)\left(2{\bar{h}}_{1}\bar{\sigma }-2{\bar{h}}_{1}+1\right)W_{0m}^{*}{\;}^{2}\\ \;\;\;\;\;\;\;\;\;\;\;+\frac{E}{4B}W_{0m}^{*}{\;}^{2}+\frac{F}{B}W_{0m}^{*}+K_{1},\;\;\;\;\;\;\;1-2{\bar{h}}_{1}p-4\bar{h}\leq W_{0m}^{*}\leq 1-2{\bar{h}}_{1}p-2\bar{h} \end{array}\\ \begin{array}{l} -\frac{D}{12B}W_{0m}^{*}{\;}^{3}+\frac{1}{2}\left[\frac{D}{2B}\left(\bar{h}+{\bar{h}}_{1}p-\frac{3}{2}\right)-\frac{C}{2B}\right]W_{0m}^{*}{\;}^{2}+\frac{\bar{h}}{B}\left(1-\bar{h}\right)W_{0m}^{*}\\ \;\;\;\;\;\;\;\;\;\;\;+\frac{D}{2B}\left({\bar{h}}^{2}-\frac{1}{4}\right)W_{0m}^{*}+K_{2},\;\;\;\;\;\;\;\;\;\;\;\;\;\;\;\;\;\;\;\;\;\;1-2{\bar{h}}_{1}p-2\bar{h}\leq W_{0m}^{*}\leq 1-2\bar{h} \end{array}\\ \frac{W_{0m}^{*}{\;}^{3}}{12B}+\frac{1}{2}\left(\frac{A}{B}-\frac{1}{2B}\right)W_{0m}^{*}{\;}^{2}+\frac{1}{4B}W_{0m}^{*}+K_{3},\;\;\;\;\;\;\;\;\;\;\;\;\;\;\;\;\;\;\;\;\;\;\;\;1-2\bar{h}\leq W_{0m}^{*}\leq 1\\ \frac{A}{2B}W_{0m}^{*}{\;}^{2}+K_{4},\;\;\;\;\;\;\;\;\;\;\;\;\;\;\;\;\;\;\;\;\;\;\;\;\;\;\;\;\;\;\;\;\;\;\;\;\;\;\;\;\;\;\;\;\;\;\;\;\;\;\;\;\;\;\;\;\;\;\;\;\;\;\;\;\;\;\;\;\;\;\;\;\;\;\;\;\;\;\;\;\;\;\;\;\;\;\;\;\;W_{0m}^{*}\geq 1 \end{array}\right.$$

where

$$\begin{array}{l} \alpha =\frac{4G^{*}\left(1-3G^{*}\right)}{3{\left(1+2G^{*}\right)}^{2}},\;U_{K}^{*}=\frac{G_{s}V_{I}{\;}^{2}}{2P_{c}b_{1}},\;W_{0m}^{*}=\frac{W_{0m}}{b_{1}},\;P_{c}=\frac{4M_{P}}{L},\\ G^{*}=\frac{G_{s}}{2G_{b}L},\;\bar{\sigma }=\frac{\sigma_{c}}{\sigma_{f}},\;\bar{h}=\frac{h}{b_{1}},\;{\bar{h}}_{1}=\frac{h_{1}}{b},\;p=\frac{b}{b_{1}},\\ A=2\bar{h}\left({\bar{h}}_{1}\bar{\sigma }+1\right)-\left(4\bar{h}+2{\bar{h}}_{1}p-1\right)\left({\bar{h}}_{1}\bar{\sigma }+\frac{2\bar{h}}{p}\right)+\bar{\sigma }{\bar{h}}_{1}p\left(1-\frac{2\bar{h}}{p}\right),\\ B=\bar{h}\left(1-\bar{h}\right)+\left[\frac{2\bar{h}}{p}+\left(1-\frac{2\bar{h}}{p}\right)\bar{\sigma }\right]\left(1-2\bar{h}-{\bar{h}}_{1}p\right){\bar{h}}_{1}p\\ \;\;+\left(1-2{\bar{h}}_{1}+2{\bar{h}}_{1}\bar{\sigma }\right)\left(1-3\bar{h}-2{\bar{h}}_{1}p\right)\bar{h}+\frac{1}{2}\left(\frac{2\bar{h}}{p}+{\bar{h}}_{1}\bar{\sigma }\right){\left(1-4\bar{h}-2{\bar{h}}_{1}p\right)}^{2},\\ C=-2\bar{h}\left(2{\bar{h}}_{1}\bar{\sigma }-2{\bar{h}}_{1}+1\right)-\left(\frac{4\bar{h}}{p}+2{\bar{h}}_{1}\bar{\sigma }\right)\left(1-4\bar{h}-2{\bar{h}}_{1}p\right),\\ D=\frac{4\bar{h}}{p}+2\bar{\sigma }\left(1-\frac{2\bar{h}}{p}\right),\\ E=\bar{h}\left(2{\bar{h}}_{1}\bar{\sigma }+1-2{\bar{h}}_{1}\right)+\left(1-4\bar{h}-2{\bar{h}}_{1}p\right)\left(2{\bar{h}}_{1}\bar{\sigma }+\frac{4\bar{h}}{p}\right),\\ F=\bar{h}\left(1-\bar{h}\right)+\left[\frac{2\bar{h}}{p}+\left(1-\frac{2\bar{h}}{p}\right)\bar{\sigma }\right]\left(1-2\bar{h}-{\bar{h}}_{1}p\right){\bar{h}}_{1}p+\frac{1}{4}\left(1-2{\bar{h}}_{1}+2{\bar{h}}_{1}\bar{\sigma }\right){\left(1-2\bar{h}-2{\bar{h}}_{1}p\right)}^{2},\\ K_{1}=\left(1-\frac{F}{B}\right)\left(1-4\bar{h}-2{\bar{h}}_{1}p\right)+\frac{1}{6B}\left(\frac{2\bar{h}}{p}+{\bar{h}}_{1}-\frac{1}{2}\right){\left(1-4\bar{h}-2{\bar{h}}_{1}p\right)}^{3}\\ \;\;-\frac{E}{4B}{\left(1-4\bar{h}-2{\bar{h}}_{1}p\right)}^{2}-\frac{1}{4B}\left(3\bar{h}-1+2{\bar{h}}_{1}p\right)\left(2{\bar{h}}_{1}\bar{\sigma }+1-2{\bar{h}}_{1}\right){\left(1-4\bar{h}-2{\bar{h}}_{1}p\right)}^{2},\\ K_{2}=\frac{4{\bar{h}}_{1}\bar{\sigma }-4{\bar{h}}_{1}-D+2}{24B}{\left(1-2\bar{h}-2{\bar{h}}_{1}p\right)}^{3}+\frac{1}{4B}\left(3\bar{h}+2{\bar{h}}_{1}p-1\right)\left(1-2{\bar{h}}_{1}+2{\bar{h}}_{1}\bar{\sigma }\right){\left(1-2\bar{h}-2{\bar{h}}_{1}p\right)}^{2}\\ \;\;-\frac{1}{4B}\left[D\left(\bar{h}+{\bar{h}}_{1}p-\frac{1}{2}\right)-C-E\right]{\left(1-2\bar{h}-2{\bar{h}}_{1}p\right)}^{2}\\ \;\;+\frac{1}{B}\left(1-2\bar{h}-2{\bar{h}}_{1}p\right)\left[F-\bar{h}\left(1-\bar{h}\right)-\frac{D}{2}{\left(\bar{h}-\frac{1}{2}\right)}^{2}\right]+K_{1},\\ K_{3}=-\frac{D+1}{12B}{\left(1-2\bar{h}\right)}^{3}+\frac{1}{4B}\left[D\left(\bar{h}+{\bar{h}}_{1}p-\frac{3}{2}\right)-C-2A+1\right]{\left(1-2\bar{h}\right)}^{2}+\frac{\bar{h}}{B}\left(1-\bar{h}\right)\left(1-2\bar{h}\right)\\ \;\;+\frac{D}{2B}\left({\bar{h}}^{2}-\frac{1}{4}\right)\left(1-2\bar{h}\right)-\frac{1}{4B}\left(1-2\bar{h}\right)+K_{2},\\ K_{4}=\frac{1}{12B}+K_{3}. \end{array}$$

in which the fully plastic bending moment *M_p_* of the rectangular sandwich tube with MFC can be given by

(A2) $$M_{p}=\sigma_{fa}bh(b_{1}-h)+\sigma_{3}bh_{1}(b_{1}-2h-h_{1})+\sigma_{2}bh(b_{1}-3h-2h_{1})+\frac{1}{4}\sigma_{1}b{\left(b_{1}-4h-2h_{1}\right)}^{2}$$

where

$$\sigma_{1}=\frac{4h}{b}\sigma_{fa}+\frac{2h_{1}}{b}\sigma_{c},\;\;\;\sigma_{2}=\frac{b-2h_{1}}{b}\sigma_{fa}+\frac{2h_{1}}{b}\sigma_{c},\;\;\;\sigma_{3}=\frac{2h}{b}\sigma_{fa}+\frac{b-2h}{b}\sigma_{c}.$$

Meanwhile, defining the impact force between the impactor and the rectangular sandwich tube as *P_r_*, the relationship between the dimensionless impact force $P_{r}^{*}$ and the dimensionless deflection $W_{0}^{*}$ of the rectangular sandwich tube with MFC under low-velocity impact can be expressed as

(A3) $$P_{r}^{*}=\left\{\begin{array}{l} \frac{3G^{*}}{3G^{*}+1}\left[\frac{1}{2B}\left(\frac{2\bar{h}}{p}+{\bar{h}}_{1}\bar{\sigma }\right)W_{0}^{*}{\;}^{2}+1\right],\;\;\;\;\;\;\;\;\;\;\;\;\;\;\;\;\;\;\;\;\;\;\;\;\;\;\;\;\;\;\;\;\;\;\;\;\;\;\;\;\;\;\;\;\;\;0\leq W_{0}^{*}\leq 1-2{\bar{h}}_{1}p-4\bar{h}\\ \begin{array}{l} \frac{3G^{*}}{3G^{*}+1}\left[\frac{W_{0}^{*}{\;}^{2}}{4B}\left(2{\bar{h}}_{1}\bar{\sigma }+1-2{\bar{h}}_{1}\right)+\frac{1}{2B}\left(3\bar{h}+2{\bar{h}}_{1}p-1\right)\left(2{\bar{h}}_{1}\bar{\sigma }-2{\bar{h}}_{1}+1\right)W_{0}^{*}\right]\\ \;\;\;\;\;\;\;\;\;+\frac{3G^{*}}{3G^{*}+1}\left(\frac{E}{2B}W_{0}^{*}+\frac{F}{B}\right),\;\;\;\;\;\;\;\;\;\;\;\;\;\;\;\;\;\;\;\;\;\;\;\;\;\;\;\;\;\;\;\;1-2{\bar{h}}_{1}p-4\bar{h}\leq W_{0}^{*}\leq 1-2{\bar{h}}_{1}p-2\bar{h} \end{array}\\ \begin{array}{l} \frac{3G^{*}}{3G^{*}+1}\left\{\frac{DW_{0}^{*}{\;}^{2}}{8B}+\left[\frac{D}{2B}\left(\bar{h}+{\bar{h}}_{1}p-\frac{1}{2}\right)-\frac{C}{2B}\right]W_{0}^{*}\left.+\frac{\bar{h}}{B}\left(1-\bar{h}\right)+\frac{D}{2B}{\left(\bar{h}-\frac{1}{2}\right)}^{2}\right\}\right.,\\ \;\;\;\;\;\;\;\;\;\;\;\;\;\;\;\;\;\;\;\;\;\;\;\;\;\;\;\;\;\;\;\;\;\;\;\;\;\;\;\;\;\;\;\;\;\;\;\;\;\;\;\;\;\;\;\;\;\;\;\;\;\;\;\;\;\;\;\;\;\;\;\;\;\;\;\;\;\;\;\;\;\;\;\;\;\;\;\;\;\;\;\;\;\;1-2{\bar{h}}_{1}p-2\bar{h}\leq W_{0}^{*}\leq 1-2\bar{h} \end{array}\\ \frac{3G^{*}}{3G^{*}+1}\left[\frac{W_{0}^{*}{\;}^{2}}{4B}+\left(\frac{A}{B}-\frac{1}{2B}\right)W_{0}^{*}+\frac{1}{4B}\right],\;\;\;\;\;\;\;\;\;\;\;\;\;\;\;\;\;\;\;\;\;\;\;\;\;\;\;\;\;\;\;\;\;\;\;\;\;\;\;\;\;\;\;\;\;\;\;\;\;\;\;\;\;\;1-2\bar{h}\leq W_{0}^{*}\leq 1\\ \frac{3G^{*}}{3G^{*}+1}\frac{A}{B}W_{0}^{*},\;\;\;\;\;\;\;\;\;\;\;\;\;\;\;\;\;\;\;\;\;\;\;\;\;\;\;\;\;\;\;\;\;\;\;\;\;\;\;\;\;\;\;\;\;\;\;\;\;\;\;\;\;\;\;\;\;\;\;\;\;\;\;\;\;\;\;\;\;\;\;\;\;\;\;\;\;\;\;\;\;\;\;\;\;\;\;\;\;\;\;\;\;\;\;\;\;\;\;\;\;\;\;\;\;\;\;\;\;\;\;W_{0}^{*}\geq 1 \end{array}\right.$$

where

$$W_{0}^{*}=\frac{W_{0}}{b_{1}},\;P_{r}^{*}=\frac{P_{r}}{P_{c}}.$$
